# Cardiovascular Care in Pediatric Cancer Survivors: Updates on Risk, Prevention, and Therapies

**DOI:** 10.1007/s11864-025-01367-9

**Published:** 2025-12-24

**Authors:** Heang M. Lim, Thomas D. Ryan, Elyse Miller, Erin Shea, Neha Bansal

**Affiliations:** 1 Pediatric Cardiology, University of Michigan, CS Mott Children’s Hospital, 1540 East Hospital Drive, Ann Arbor, MI 48109-4204 USA; 2 Department of Pediatrics, University of Cincinnati College of Medicine, Heart Institute, Cincinnati Children’s Hospital Medical Center, Cincinnati, OH USA; 3 Pediatric Cardiology, Le Bonheur Children’s Hospital, University of Tennessee Health Science Center, Memphis, TN USA; 4 Pediatric Cardiology, Duke University Medical Center, Durham, NC USA; 5 Pediatric Cardiology, Mount Sinai Kravis Children’s Hospital, New York, NY USA

**Keywords:** Pediatric cardio-oncology, Childhood cancer survivors, Cardiotoxicity, Cardioprotection, Survivorship care

## Abstract

Improved survival in pediatric oncology has highlighted the growing burden of cancer treatment-related cardiotoxicity among survivors of childhood cancers. While the cardiotoxicity of anthracyclines and chest radiation are well documented as major contributors of late morbidity and mortality, the rapid adoption of immunotherapies and targeted agents in pediatrics raises new concerns regarding the unknown long-term cardiovascular risk. Recent advances include risk-adapted surveillance protocols and pediatric-based imaging guidelines are important steps towards optimizing early detection and intervention. However, significant gaps persist, particularly in the development of effective treatment options for cardiotoxicity, consistent cardiovascular event reporting, seamless transitions to adult care, and the meaningful integration of machine learning and precision medicine into real-world practice. In light of these ongoing challenges, we believe that while national and international guidelines are an essential framework, optimal cardiology care for pediatric cancer survivors must be grounded in individualized assessment and supported by multidisciplinary collaboration. Ongoing research and innovation are imperative to closing these gaps and advancing the long-term cardiovascular outcomes of this vulnerable population.

## Introduction

Cancer treatment-related cardiotoxicity (CTRC) has emerged as a leading cause of morbidity and premature mortality among survivors of pediatric cancer. With the 5-year survival rates for childhood cancer now exceeding 80%, the long-term sequelae of cancer therapy have become a central focus of survivorship with increasing recognition of the need for lifelong multidisciplinary care to optimize cardiovascular health [1–5]. Childhood cancer survivors (CCS) have an 8- to 10-fold higher rate of cardiac mortality compared to age-matched controls, and nearly one-third will develop a severe or life-threatening chronic health condition within 20 years of diagnosis [2, 4].

The pathogenesis of CTRC is multifactorial, involving direct myocardial injury, vascular damage, and the acceleration of traditional cardiovascular risk factors such as hypertension, dyslipidemia and diabetes [1, 3, 6]. Anthracycline chemotherapy and chest-directed radiation therapy, both of which are integral to the treatment of many pediatric malignancies, are well established contributors to CTRC [2, 4, 6–8]. The increasing use of novel targeted agents and immunotherapies in the pediatric population has introduced new patterns of cardiotoxicity with unknown long-term implications [1, 6]. In the era of precision medicine, genomic studies have identified polymorphisms in drug metabolism and cardioprotective pathways that may modify individual susceptibility to CTRC and offer more individualized protection strategies [1, 2, 6]. Ongoing trials are evaluating the efficacy of primary and secondary prevention strategies, including the use of neurohormonal antagonists, statins, and structured exercise programs [2, 6]. This review highlights recent advances and emerging priorities in cardiology care for CCS.

## Cardiotoxicity

### Conventional Cancer Therapies

Anthracyclines, including doxorubicin, daunorubicin and epirubicin, remain among the most effective antineoplastic agents in pediatric oncology, used in nearly 60% of childhood cancer protocols [4]. Despite their clinical efficacy, anthracyclines are associated with a dose-dependent, progressive, and often irreversible cardiomyopathy. Subclinical changes in cardiac structure and function can be detected within years of exposure, with up to 50% of survivors demonstrating echocardiographic abnormalities and 16% developing symptomatic heart failure [3, 4]. The risk of heart failure increases with cumulative dose, younger age at exposure, female sex, and concomitant chest radiation [2, 4, 5, 7–9]. Other agents, such as mitoxantrone, high-dose cyclophosphamide, and ifosfamide, have also been implicated in late cardiac dysfunction, though often to a lesser severity [4].

Chest-directed radiation therapy is a potent risk factor for a spectrum of CVD involving the coronary arteries, valves, pericardium, and conduction system [2–4, 6, 8]. The risk is dose-dependent, synergistic with anthracycline exposure, and more pronounced in young patients. The Pediatric Normal Tissue Effects in the Clinic (PENTEC) initiative has demonstrated that each 10-Gy increase in mean cardiac dose nearly doubles the hazard for coronary artery disease, heart failure, and valvar disease [8]. Advances in radiation delivery techniques have reduced cardiac exposure, but long-term surveillance remains essential [2, 8].

### Emerging Cancer Therapies

While cardiotoxicity has been linked to new chemotherapeutics across patient populations, most published reports have focused on adults, despite the growing use of these agents in pediatric oncology. Although cardioprotective strategies exist for anthracycline toxicity, no such measures have been developed for newer agents.

Immune checkpoint inhibitors (ICI) have been associated with cardiac arrhythmia, other electrocardiogram changes, myocarditis and/or pericarditis [5]. Although the incidence of myocarditis is low (estimated to be 0.1–1.2%), when it does occur, it can be fatal in 25–50% of cases [10]. In contrast, a single center study completed by Mirabel et al. examined patients prior to and after ICI administration and found no cases of myocarditis or cardiovascular-related deaths, but more commonly found electrophysiologic changes and heart failure that did not warrant hospital admission [11].

Although mitogen-activated protein kinase (MEK) inhibitors are more commonly used in the adult population [12], there is growing use of it in pediatric gliomas [13], which accounts for nearly one third of pediatric central nervous system tumors [14]. In adults, MEK inhibitor therapy has been associated with cardiotoxicities including decreased left ventricular ejection fraction (LVEF), hypertension, pericarditis, and cytokine release syndrome (CRS) [15]. Although rare, QTc prolongation with MEK inhibitors can be seen in adults especially when used in combination with BRAF inhibitors [16]. Although MEK inhibitor use is rare in pediatrics, CTRC has been seen, but data are limited to single-center retrospective studies. Bender et al. observed that 28% of patients were found to have dysfunction (LVEF < 55% and/or a decrease ≥ 10%) with subsequent improvement when the MEK inhibitor was either held or treatment was completed [17]. Robison et al. also observed a high rate of occurrence, with nearly 50% of their cohort experiencing a decline in LVEF [13]. Despite the observed decline in ejection fraction, neither study reported any cases of symptomatic heart failure.

Even more severe can be CRS related to chimeric antigen receptor (CAR) T-cell therapy which can trigger a state of shock as well as electrophysiologic alterations, myocardial dysfunction, and cardiac arrest [5]. Shalabi et al. noted the majority of children and young adults who developed CRS had pre-treatment baseline echocardiogram abnormalities [18]. Those with CRS and an abnormal troponin level, developed left ventricular (LV) systolic dysfunction (either new or > 10% decrease from baseline). 12% of the cohort had a decrease in LV systolic function with CRS, and two-thirds of those had full recovery of their LVEF to normal by 28 days post- CAR T-cell therapy. The remaining patients showed improvement in their LVEF to baseline within three months [18].

Bispecific T-cell engager antibodies, such as blinatumomab, have also been linked to myocarditis, pericarditis, conduction abnormalities, coronary disease, myocardial infarction, and heart failure in the adult population [19]. While the use of blinatumumab in pediatric oncology is still emerging, recent phase 3 trials have demonstrated improved disease-free survival when used in combination with chemotherapy [20]. However, the cardiotoxic effects of bispecific T-cell engager antibody therapy in the pediatric population remain largely unexplored.

It is important to note that most publications exploring the cardiotoxic effects of these novel agents are based on adult populations who have much higher rates of pre-existing cardiovascular disease (CVD) risk factors than pediatric patients. Often, these studies have short follow-up durations, limiting the ability to evaluate long-term cardiovascular outcomes in cancer survivors. In addition to their acute cardiotoxic effects, these newer agents may also contribute to secondary comorbidities such as hypertension and diabetes, both of which are well-established risk factors that significantly increase long-term CVD in certain cancer survivors [21].

## Surveillance and Risk Assessment of Cardiotoxicity

### Updated Surveillance Guidelines

Recognizing the heterogeneity of cardiovascular risk among survivors, recent efforts have focused on individualized risk stratification to guide surveillance and preventive strategies. The International Late Effects of Childhood Cancer Guideline Harmonization Group (IGHG), the American Society of Echocardiography (ASE), and the Children’s Oncology Group (COG) have all published risk-based guidelines that incorporate cumulative anthracycline dose, chest radiation dose, age at exposure, and other clinical factors [2, 7, 22].

The IGHG published a systematic review evaluating the benefits, harms and cost-effectiveness of cardiomyopathy surveillance in childhood cancer survivors which led to updated recommendations [7]. Among the notable changes, the radiation dose threshold for high-risk survivors was lowered from ≥ 35 Gy to ≥ 30 Gy. Previously, individuals who were classified as low risk (those who received < 100 mg/m2 of anthracyclines and/or < 15 Gy of chest radiation) were recommended to undergo cardiomyopathy surveillance every five years. In the updated guidelines, routine screening is no longer recommended for this group, based on the low incidence of heart failure and the lack of cost-effectiveness of routine screening.

Following the IGHG updated recommendations, the COG released the latest revisions to the Long Term Follow-Up Guidelines [22]. Overall, the recommendations for cardiac screening for low-risk survivors aligned with those of the IGHG, eliminating routine screening for those at low risk for CRTC.

Both IGHG and the COG recommendations for cardiomyopathy screening continue to heavily rely on 2D echocardiography, which remains the primary imaging modality for assessing cardiac function in children undergoing cancer treatment [7, 22]. Its non-invasive nature and broad availability make it easy for widespread use in pediatrics. Since changes in echocardiographic findings can influence whether a patient continues with their current chemotherapy regimen, having a standardized protocol to ensure consistent treatment and surveillance is a top priority. The American Society of Echocardiography (ASE) has published recommendations on the use of echocardiographic parameters, with particular emphasis on maintaining consistent methodology for serial assessments of ejection fraction and, when available, global longitudinal strain [2]. Preferably, the biplane Simpson method should be used, with the area-length as a reasonable alternative if apical 2 chamber views are not optimal. Additionally, LV volumetric diastolic measurements should be considered, including assessment of left atrial volume for assessment of diastolic dysfunction. When uniformly adopted, these recommendations are expected to enhance the standardization of functional cardiac assessment across centers caring for CCS [2].

In addition to these updated surveillance recommendations, the American Heart Association (AHA) continues to emphasize the importance of individualized cardiovascular screening based on clinical assessment, particularly in the presence of symptoms or additional risk factors [4, 5, 9].

### Risk Assessment

The St. Jude/Chow risk prediction model represents a major advance in personalized risk assessment, integrating demographic, treatment, and genetic variables to estimate an individual’s long-term risk of heart failure and other cardiac outcomes [1, 6]. These models facilitate shared decision-making regarding the intensity of surveillance, the use of cardioprotective interventions and the management of modifiable risk factors. Analyses regarding cost-effectiveness have demonstrated that routine echocardiographic screening is justified for moderate- and high-risk survivors but not for those at lowest risk, who constitute approximately 40% of the survivor population [2, 5, 7, 9].

## Prevention and Strategies for Risk Mitigation

### Dexrazoxane

Dexrazoxane remains the only Food and Drug Administration (FDA)- approved agent for primary prevention of anthracycline-induced toxicity. In 2014, dexrazoxane was granted the orphan drug designation by the FDA for use in pediatric patients up to 16 years of age receiving anthracycline chemotherapy [23]. Various pediatric trials have established that dexrazoxane is effective in preventing serious cardiovascular complications, including systolic dysfunction and heart failure [24–27]. Chow et al. revealed that dexrazoxane administration was associated with better systolic function and lower risk of reduced ejection fraction [28]. This protective effect was most evident in patients treated with cumulative doxorubicin doses of ≥ 250 mg/m2, with observed benefits even up to 20 years after exposure [28]. Among patients who received dexrazoxane, the rates of serious cardiac complications were extremely low, with no patients in the treated cohort who required heart transplantation, which included individuals receiving even > 600mg/m^2^ of doxorubicin [29].

There were concerns that the use of dexrazoxane was associated with an increased risk of secondary malignant neoplasms [30, 31]. However, results from mid- and long-term follow-up studies have not shown an association with dexrazoxane and increased risks of secondary malignancies, death, or serious cardiovascular events [29, 32]. The European Medicines Agency also found data that align with this, and in 2017, a statement was issued that removed the contraindication for dexrazoxane use in those who have received high dose anthracycline [33]. When the risk of cardiac damage is anticipated to be high, dexrazoxane use may be justified in children receiving anthracyclines [34]. As there is no safe cumulative dose of anthracycline, and recent data dispelling earlier concerns about potential harmful effects of dexrazoxane, some groups are advocating for its use prior to every anthracycline dose as a strategy for primary prevention of related cardiomyopathy [35, 36].

In 2022, the IGHG developed a global guideline for dexrazoxane use in children with cancer who are expected to receive anthracyclines [37]. They concluded that the benefits of dexrazoxane likely outweighed the potential risk of secondary neoplasms when the cumulative doxorubicin (or equivalent) dose is at least 250mg/m^2^ [37]. No recommendation was made for patients receiving cumulative doxorubicin dose ≤ 250mg/m^2^ since the evidence was insufficient [37]. However, the recent AHA scientific statement regarding treatment strategies for cardiomyopathy in children supports the use of dexrazoxane starting with the first dose of anthracycline [36].

### Genetic/Genomic Associations and Biomarkers

Genetic variation has emerged as a critical factor influencing individual susceptibility and development of cardiotoxicity associated with chemotherapy. A recent systematic review identified several variants associated with cardiomyopathy after treatment with anthracycline and chest radiotherapy [38]. The SLC28A3 rs7853758 AA + GA genotype was detected more frequently in patients who did not develop cardiotoxicity and was significantly lower in the group with cardiotoxicity [39]. Specifically looking at adolescent/young adults (AYA) cancer patients, as opposed to CCS, several variants were associated with cardiac dysfunction: *SLC10A2*:rs731998, *SLC22A17*: rs4982753, *HAS3*:rs2232228, which was noted to be opposite to the findings in CCS, insinuating that the genetic influence on cardiac dysfunction in AYA may be different than those in childhood cancer survivors [40]. Additional genes attributed to anthracycline-induced cardiotoxicity included CBR2, CELF4, GSTM1, HAS3, RARG, and SLC28A3, and additional findings of HNMT and SLC22A2 in younger children, ABCB4 in females, and SULT2B1 in males [41].

Differential mRNA expression profiles in peripheral blood of anthracycline-exposed CCS may indicate increased sensitivity to anthracycline and thus higher likelihood of developing cardiomyopathy [42]. Elevated lactate dehydrogenase A may suggest metabolic changes in a failing heart [42], and dysregulation of proinflammatory cytokine receptors such as IL1R1and IL1R2 may be linked to manifestations of symptomatic cardiotoxicity. Leptin may be an emerging biomarker for stress and has been shown to be higher in CCS exposed to anthracycline treatment compared to controls and associated with adverse vascular changes [43, 44].

Lipshultz et al. studied the C282Y and H63D mutations of the hemochromatosis gene (HFE), which is associated with hereditary hemochromatosis [45]. Since anthracyclines bind to iron in the blood to form free radicals, which injure or kill cardiomyocytes, they hypothesized that children with higher iron levels, such as those with HFE gene mutations may have higher cardiomyocyte injury. In 184 survivors of childhood acute lymphoblastic leukemia, after controlling for dexrazoxane treatment, the 10% of patients heterozygous for C282Y, had nearly a nine-fold increased incidence of elevated cTnT concentrations, suggesting myocardial injury, compared to those without this HFE gene mutation. Those patients with C282Y mutations had significantly more abnormal LV structure and function by echocardiography 2 years later than those without this HFE mutation [45].

Advancements in genomic research offer the potential to improve identification of individuals at greatest risk for CTRC. The ability to incorporate routine genetic screening into clinical practice would enable detection of patients with a genetic predisposition to cardiotoxicity, allowing clinicians the opportunity to personalize chemotherapy regimens to minimize cardiac risk while maintaining efficacy. Furthermore, genetic screening would identify high-risk individuals who may benefit from more frequent cardiac surveillance than currently recommended for the general population. This individualized approach to survivorship care would facilitate earlier detection and management of cardiotoxicity in those most susceptible.

### Exercise and Rehabilitation

Exercise should be considered integral through all phases of cancer care and is generally considered safe for those survivors without cardiomyopathy. Prescriptions for exercise during pediatric cancer therapy and survivorship should be individualized and adjusted for baseline cardiopulmonary fitness and functional impairment with consideration of guidance from a cardiologist for those with cardiomyopathy [5].

In a survey, among CCS, only 34% fulfilled the World Health Organization physical activity recommendation of at least 60 min of daily moderate-to-vigorous physical activity with prolonged sedentary time of over 8 hours a day [46]. This is in contrast to 72% of adult cancer survivors meeting criteria for adequate physical activity [47]. Risk factors for increased sedentary lifestyle included being female sex, older age, overweight, survivors of central nervous system tumors, or experiencing relapse [46]. Young adult survivors of pediatric hematopoietic stem cell transplant have an increased risk of reduced physical capacity with nearly 28% fulfilling criteria for metabolic syndrome [48]. Additionally, CCS have been shown to have higher body mass indices [49, 50] and consistently lower peak VO_2_ [51, 52]. In ALL and Hodgkin lymphoma CCS, lower peak VO2 was found in the setting of normal LVEF and global longitudinal strain, suggesting overall baseline deconditioning in this cohort [51].

Systematic reviews evaluating a variety of lifestyle and physical activity interventions for pediatric and AYA with cancer showed mixed results regarding efficacy, however, the majority reported at least partial improvements in physical activity [53], fatigue, muscle strength, aerobic capacity, physical functioning [54]. In a randomized controlled trial of CCS, a one-year physical activity intervention was found to reduce CVD risk scores across all analyses [55], with dose-graded aerobic exercise training alongside physical rehabilitation improving cardiopulmonary fitness in pediatric ALL survivors [56]. Long-term exercise intervention in children with acute lymphoblastic leukemia (ALL) during treatment and consolidation phases significantly improved endurance, mobility, cancer-related fatigue, and quality of life, and muscle strength [57]. Currently, the Adapted Physical Activity for Children treated for Cancer and Insulin-Sensitivity study is investigating the metabolic and physical effects of low- versus high-intensity exercise programs initiated as early as the time of cancer diagnosis [58].

Despite these benefits, access to exercise services and cancer rehabilitation remain lacking. Providers surveyed all strongly agreed that cancer rehabilitation was important to improving patient outcomes, however, barriers included insurance coverage, lack of standardized screening and referral patterns and inconsistent patient participation [59]. One approach to address the lack of cancer rehabilitation services is through a multidisciplinary family lifestyle intervention, which has been shown to increase the total minutes spent in physical activity [60]. Additionally, in a study of CCS and caregiver dyads, caregiver activity level was the strongest predictor of CCS activity level, suggesting that behavioral interventions to increase physical activity should target both the caregiver and CCS [50].

### Oral Therapies for Cardiotoxicity

The use of oral heart failure therapies for primary prevention in pediatrics of treatment-related cardiotoxicity is not supported by current evidence [36]. Although a few randomized controlled trials have investigated agents for secondary prevention, the evidence remains limited and insufficient to support routine use [5]. The ACE inhibitor After Anthracycline study was an early randomized, double-blind, controlled trial which compared enalapril with placebo in pediatric cancer patients which revealed that enalapril reduced LV end systolic wall stress. However, side effects from enalapril included dizziness and hypotension, with no positive impact on exercise performance [61, 62]. A subsequent randomized, double-blind controlled trial in patients with leukemia and lymphoma compared enalapril to placebo over a six-month period [63]. While both groups experienced a decline in LVEF, the reduction was more pronounced in the placebo group, which also had higher levels of pro-BNP and cardiac troponin I. These findings suggest that enalapril may play a role in mitigating anthracycline-induced cardiotoxicity however, the short follow-up period warrants cautious interpretation.

Most recently, PREVENT-HF, a randomized, controlled phase 2 trial, comparing carvedilol to placebo in CCS ≥ 40 kg with an LVEF > 50% and/or LV fractional shortening > 25% found that low-dose carvedilol was safe for those at risk of heart failure but did not improve LV wall thickness-dimension ratio Z-scores and so results did not support its use [64].

No large randomized controlled trials have identified effective therapies for stage C heart failure due to CRTC, especially in pediatrics [36]. In clinical practice, pediatric cardiologists and cardio-oncologists often rely on heart failure medications with demonstrated benefits in adults, while acknowledging the limited evidence supporting their use in pediatrics. Although guidelines exist for heart failure management in pediatrics with a variety of cardiomyopathies, they are not specific to those with CTRC [65, 66], and significant practice variability remains among centers [67].

Emerging adult data suggests that SGLT2 inhibitors may play a role in mitigating CTRC with some studies demonstrating improved cardiac outcomes in patients [68–70]. However, evidence surrounding the use of SGLT2 inhibitors for CTRC is lacking, and its safety and efficacy in children have yet to be determined [36, 71].

In adults, the STOP-CA trial demonstrated that initiating daily prophylactic atorvastatin prior to anthracyclines significantly reduced the percentage of patients who experienced a ≥ 10% decline in LVEF compared to those on placebo [72]. However, no such study of statins exists in CCS.

## Future Directions and Unmet Needs

### Social Determinants of Health

Cardiovascular disease outcomes are known to be affected by systemic, structural and psychosocial factors although the exact interplay between these factors is understudied [73] and should be considered in the risk-based assessment [74, 75]. The recent AHA Scientific Statement acknowledges that social determinants of health influence both long term cardiovascular risk and cancer survival, underscoring the need to expand disparities research, enhance social needs screening, improve bias reduction training, and increase policy-level engagement [76]. However, how social determinants of health specifically affect long-term outcomes in CCS remains poorly understood. Findings from the St. Jude Lifetime Cohort Study revealed that childhood cancer survivors who live in a US census block with high area deprivation index, which is associated with neighborhood-level socioeconomic disadvantage, had an increased risk of late mortality [77]. CCS maybe be particularly vulnerable, as they often have limited understanding of their prior cancer treatment and inadequate awareness of potential late effects [78]. This vulnerability is compounded by a higher risk of being lost to follow-up and poor adherence to recommended surveillance protocols [76, 79].

### Improving Reporting of Cardiovascular Events and Safety in Childhood Cancer Survivors

Reporting of cardiovascular events in CCS is essential to understanding long-term risks, informing surveillance guidelines, and advancing cardioprotective strategies. However, current reporting practices remain highly variable, often limited by inconsistent definitions, underreporting, and a lack of standardized cardiovascular safety endpoints. A major challenge lies in the heterogeneity of how cardiovascular outcomes are defined and graded across studies, hindering meaningful comparisons and analyses. Most definitions are not optimally designed to capture subtle or early cardiac dysfunction, which limits the ability for early identification of at-risk individuals. Recent efforts to develop more nuanced grading systems that include subclinical measures (e.g., abnormal echocardiographic strain or elevated biomarkers) are promising but require broader validation and implementation [80, 81].

Another barrier to accurate reporting is under-diagnosis, especially in long-term follow-up settings where survivors may not receive regular cardiovascular screening. In a cross-sectional study including 293 survivors, primary care physician records had infrequent documentation referencing a cancer history (67.6%) or a need for late-effects surveillance (32.4%), and only 21.5% of records had a completed or planned echocardiogram in the prior 2 years [81]. Embedding standardized cardiac assessments and increasing awareness of lifelong surveillance can improve event detection. Furthermore, linking electronic health records (EHR) with cancer registries and cardiology databases can facilitate real-time surveillance and retrospective analysis of cardiovascular events [82].

To improve cardiovascular safety, longitudinal studies and pharmacovigilance registries are needed to capture late-onset cardiotoxic effects of newer therapies, including targeted agents and immunotherapies with distinct cardiac profiles compared to traditional chemotherapies. Additionally, the inclusion of patient-reported outcomes and functional assessments (e.g., exercise capacity) can provide a more holistic view of cardiovascular health in survivors.

### Transition of Pediatric Childhood Cancer Survivors to Adult Cardio-Oncology Care

As the population of CCS grows, more survivors enter adulthood with long-term risks of cardiovascular complications. As pediatric cardiologists and oncologists are not experts in managing adult comorbidities, transition to adult providers is ultimately necessary. A well-coordinated transition from pediatric to adult cardio-oncology care is critical to ensuring lifelong cardiovascular surveillance and risk management. In 2002, the COG established the Survivorship Transition Task Force as a component of its Adolescent/ Young Adult Committee [83]. The COG and the AHA both recommend initiating transition planning as early as age 12, with a formal transfer to adult services ideally occurring between ages 18 and 21. Studies show that many CCS are lost to follow-up during this transition period due to multiple barriers including insufficient education about late effects, insurance changes, psychosocial challenges, and a lack of adult providers experienced in cancer survivorship care [80].

The transition process involves more than the physical transfer of care and must be a structured, patient-centered approach that promotes medical autonomy, treatment understanding, and consistent follow-up. Several models exist for transitioning care, and the optimal approach depends on multiple factors including the types of care centers, both cancer and primary care centers, available within a given region. For many, a hybrid model with ongoing interactions between both cancer and primary care centers may be the most effective option [84]. There is no single model of care that is “best” for all settings. However, for successful transitions, continued collaboration between adult and pediatric providers is crucial and the process should be tailored to the individual needs of each survivor [85]. Digital innovations such as survivorship care plans, mobile health apps, and patient portals also hold promise in empowering survivors to track appointments, medications, and symptoms during the transition phase [86].

Despite these advances, major gaps remain. There is a pressing need for evidence-based transition protocols, increased training for adult providers in pediatric-onset conditions, and systemic policy changes to support continuous care.

### Use of Artificial Intelligence for Detection and Prediction of Cardiotoxicity

Artificial intelligence is emerging as a powerful tool in medicine as well as cardio-oncology [87]. Machine learning algorithms can process vast datasets including images, biomarkers, electrocardiograms, and electronic health records (EHRs) to identify patterns predictive of LV dysfunction before clinical symptoms manifest [88–90]. Artificial intelligence-enhanced image analysis has shown promise in improving the sensitivity of echocardiographic LV function measurements to recognize subtle changes [90].

Edwards and colleagues had a “proof of concept” machine learning-assisted tool to predict children at risk of CTRC using echocardiographic images as the primary input employing Deep Convolutional Neural Network [91]. In their study, the network was able to predict which individuals would go on to develop cardiomyopathy, especially when more echocardiograms were included. Moreover, predictive models incorporating clinical factors such as age, gender, anticipated cumulative anthracycline dose, radiation exposure, and genetic polymorphisms have demonstrated improved risk stratification using machine learning techniques [92]. While these advancements are promising, ethical considerations, model interpretability, and clinical validation remain critical challenges before widespread implementation in pediatric cardio-oncology.

## Conclusion

Pediatric cancer survivors are at an increased risk for long-term complications, with cancer-treatment related cardiotoxicity representing a significant contribution to late morbidity and mortality. Despite growing awareness, effective strategies for both the prevention and treatment of cardiotoxicity in the pediatric population remain limited with current strategies largely extrapolated from adult data. There is a pressing need to continue multidisciplinary collaboration and expand research to identify effective strategies that reduce cardiovascular risk, both prior to and following exposure to cardiotoxic chemotherapy, and ultimately improving long-term outcomes for pediatric cancer survivors into adulthood.

## Key References

- Mertens L, Singh G, Armenian S, Chen MH, Dorfman AL, Garg R, et al. Multimodality Imaging for Cardiac Surveillance of Cancer Treatment in Children: Recommendations From the American Society of Echocardiography. J Am Soc Echocardiogr. 2023;36(12):1227-53.
  - This consensus guideline is of outstanding importance as it is the first document to provide recommendations for standardized imaging modalities and details the methodology for cardiac monitoring in pediatric cancer patients
- Ryan TD, Bates JE, Kinahan KE, Leger KJ, Mulrooney DA, Narayan HK, et al. Cardiovascular Toxicity in Patients Treated for Childhood Cancer: A Scientific Statement From the American Heart Association. Circulation. 2025;151(15):e926-e43.
  - This reference is of outstanding importance as it provides the most up-to-date consensus and expert guidance regarding the latest in cardiovascular care for the pediatric cancer survivor, highlighting both clinical practice and research priorities
- Ehrhardt M, Leerink JM, Mulder RL, Mavinkurve-Groothuis A, Kok W, Nohria A, et al. Systematic review and updated recommendations for cardiomyopathy surveillance for survivors of childhood, adolescent, and young adult cancer from the International Late Effects of Childhood Cancer Guideline Harmonization Group. Lancet Oncol. 2023;24(3):E108-E20.
  - This reference is of outstanding importance as it is the definitive international guideline for childhood cancer survivors and sets the standard for cardiac surveillance in this patient population

## Data Availability

No datasets were generated or analysed during the current study.
